# Non-coding RNAs as predictive factors of oncological outcomes in plasma cell myeloma

**DOI:** 10.3389/or.2026.1868835

**Published:** 2026-08-14

**Authors:** Alicja Bogdanowicz-Żeleźniak, Natalia Moniczewska, Maciej Korpysz, Aneta Szudy-Szczyrek, Radosław Mlak

**Affiliations:** 1 Department of Laboratory Diagnostics, Medical University of Lublin, Lublin, Poland; 2 Doctoral School, Medical University of Lublin, Lublin, Poland; 3 Department of Biochemical Diagnostics, Medical University of Lublin, Lublin, Poland; 4 Department of Hematooncology and Bone Marrow Transplantation, Medical University of Lublin, Lublin, Poland

**Keywords:** diagnosis, non-coding RNA, plasma cell myeloma, predictive factors, prognosis

## Abstract

Plasma cell myeloma (PCM) is the second most common haematological malignancy and accounts for 1% of all hematological tumors. PCM remains a challenging disease to treat because of its heterogeneity. Despite therapeutic advances, a significant proportion of patients do not respond to treatment or develop resistance, highlighting the need to identify reliable predictive biomarkers. Recent research suggests that non-coding RNAs (ncRNAs), i.e., microRNA (miRNA), long non-coding RNA (lncRNA) and circular RNA (circRNA) are important regulators of gene expression and play a key role in the pathogenesis. Epigenetic changes precede genetic and protein modifications, and the analysis of a single ncRNA can reflect the status of several genes. Moreover, ncRNAs are stable and can be detected in a minimally invasive manner using readily available biological samples. This review synthesizes current preclinical and clinical data on ncRNAs as predictive biomarkers of response to PCM therapies, identifies the most promising molecules and their mechanisms of action, highlights key methodological limitations, and proposes a translational validation framework necessary for clinical implementation. The most promising candidates are miR-21, lncRNA: MALAT1, and PCAT-1, which are well established in the literature and confirmed at both the mechanistic and clinical levels. However, small cohorts, heterogeneity of studies, and lack of methodological standardization limit the strength of this evidence, highlighting the need for prospective multicenter studies with standardized analytical procedures.

## Introduction

Despite the enormous progress made in recent years in the treatment of PCM, particularly in terms of the availability of new molecularly targeted drugs, a significant proportion of patients still do not respond or develop resistance to the treatment, leading to poor outcomes. One of the key elements determining effective treatment is the precise determination of predictive factors, defined as providing information on a patient’s response to a specific drug or treatment regimen, and prognostic factors, defined as providing information on the course of the disease regardless of treatment (1,2). In particular, predictive factors allow for the personalization/individualization of therapy, which ultimately enables further improvement in treatment outcomes. Although the catalogue of prognostic factors is continuously expanding, reliable predictive factors remain largely unavailable in PCM (3). Therefore, owing to the significant heterogeneity of treatment responses and the often ambiguous results of studies on predictive factors in PCM, there is an urgent need to systematize the available knowledge in this field (1).

Increasing evidence suggests that selected ncRNAs, particularly miRNAs, may serve as promising candidates pending validation (2–4). Equally important, epigenetic changes may precede changes at the genetic or protein level (4).

The first reports suggesting the potential usefulness of selected ncRNAs (especially miRNAs) as PCM biomarkers concerned the significant involvement of these molecules in the pathomechanism of this disease (3,5,6). For example, miR-155, which downregulates the expression of *PSMB5*, which encodes the β5 subunit of the proteasome (PSMβ5), a direct binding site for proteasome inhibitors (PIs), leading to reduced proteasome activity and increased sensitivity of PCM cells to bortezomib (7). In turn, reduced expression of lncRNA PCAT-1 negatively regulates the Bcl-2 family of antiapoptotic genes, and its inhibition increases the efficacy of bortezomib therapy (8). A similar effect of increased sensitivity to bortezomib was observed after silencing circ_0007841, which was associated with a decrease in *JAG1* gene expression (due to the unblocking of miR-129-5p) and the inhibition of PCM cell proliferation (9).

**FIGURE 1:**
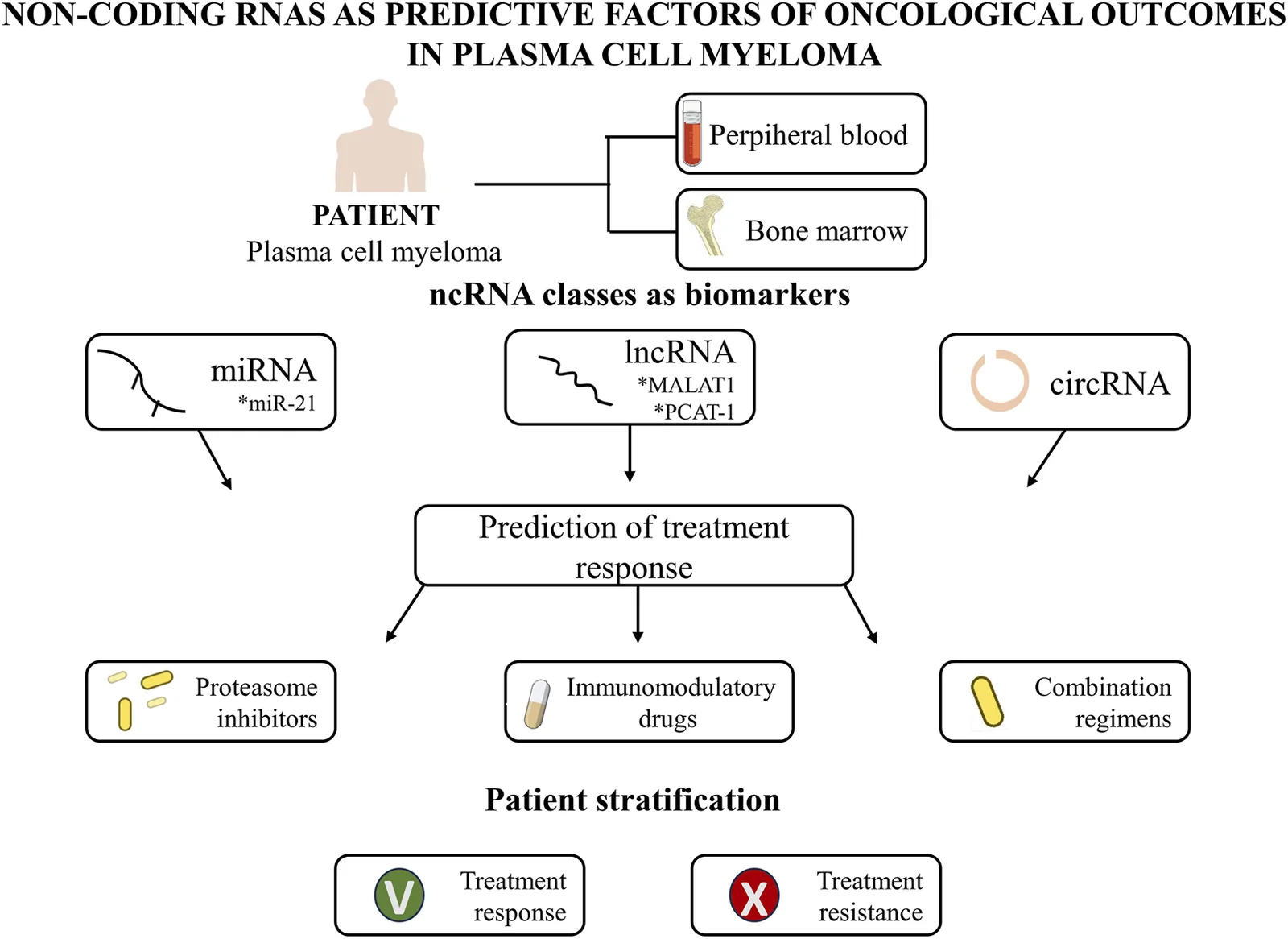
NcRNAs in PCM treatment response prediction.

Therefore, the primary purpose of this review is to: (1) critically synthesize and evaluate the current evidence from preclinical and clinical studies on ncRNAs as predictive biomarkers of response to key PCM therapies (PIs and immunomodulatory drugs (IMiDs)); (2) identify the most promising candidate ncRNAs and their mechanisms of action; (3) highlight the major methodological limitations and inconsistencies in the field; and (4) propose a framework for the translational validation required to move these biomarkers toward clinical utility. The concept of the study was demonstrated on Figure 1.

PCM is the second most common haematological cancer, accounting for 1% of all cancers. It remains a great challenge because of its high incidence (∼4/100,000 people/year) and mortality (1.8/100,000 people/year) (10,11).

The progression of PCM is often preceded by an asymptomatic stage known as monoclonal gammopathy of undetermined significance (MGUS) or a more advanced stage known as smouldering multiple myeloma (SMM). Risk factors icluding ionizing radiation, pesticides, chronic inflammation, and a positive family history (12,13). Cytogenetic abnormalities, inculding immunoglobulin heavy chain (IgH) translocations, trisomies, monosomy 13, deletions - del(13q), del(17p), del(1p), duplication (1q21), *MYC* translocations, and hypodiploidy (14).

PCM diagnosis is based on the International Myeloma Working Group (IMWG) SLiM-CRAB criteria (15). While the SLiM-CRAB criteria allow for the diagnosis of PCM, prognosis assessment is based on the revised international staging system (R-ISS) and the second revision of the international staging system (R2-ISS). However, currently available risk stratification systems cannot reliably predict response to specific therapies, highlighting the need for novel predictive biomarkers such as ncRNAs (16).

The introduction of new therapies for the treatment of PCM has significantly improved the long-term survival. Current treatment includes PIs, IMiDs, monoclonal antibodies (mAbs), bispecific antibodies (BsAbs), adoptive cell transfer therapy (CAR-T) and autologous hematopoietic stem cell transplantation (ASCT).

However, despite significant progress in this field, some patients with PCM can develop resistance to the therapies used, which ultimately leads to treatment failure. Therefore, it is essential to conduct further research to eliminate all difficulties associated with the selected treatment method and identify factors that will allow predicting the response to the used therapy (17–31).

Significant advances in treatment have led to an increased rate of complete remission (CR) in patients with PCM. However, despite achieving CR, some patients may experience relapse or PCM progression (31). Therefore, sensitive methods for evaluating treatment response are essential. Minimal residual disease (MRD) has become one of the most sensitive indicators of treatment response and prognosis (32–34). MRD is currently assessed using multiparameter flow cytometry (MFC) or next-generation sequencing (NGS), with a sensitivity of approximately 10^−5^ to 10^–6^ (35).

In addition, positron emission tomography (PET-CT) and circulating tumor-derived free DNA (ctDNA) in peripheral blood (PB) demonstrate great potential in MRD assessment and early recurrence detection (36–40).

The prognosis of PCM depends on many factors, including individual patient characteristics, the presence of cytogenetic changes, disease stage, and response to treatment. Although therapeutic advances have substantially improved survival outocomes, prognosis remains heterogeneous among patients (1,41). This variability further emphasizes the need for reliable predictive biomarkers that would enable individualized treatment selection and improve clinical outcomes (1).

## Methodology

This review was designed as a narrative review with a structured literature search and study selection workflow informed by PRISMA principles.

The literature search covered the years 2017–2024 and was conducted in electronic databases such as PubMed, Scopus, and Google Scholar, using the following defined keywords: ‘multiple myeloma’, ‘plasma cell myeloma’, ‘ncRNA’, ‘miRNA’, ‘lncRNA’, “circRNA”, ‘response to treatment’, ‘therapy’, ‘drug resistance’, ‘drug sensitivity’, ‘predictive marker’.

The review included publications available in full-text, without language restrictions, provided they contained sufficient information to assess their relevance to the subject of the review. Inclusion criteria included: 1) studies directly related to the role of ncRNAs in PCM; 2) studies addressing predictive value in the context of treatment response; 3) both clinical and preclinical studies; 4) original articles and review articles. Exclusion criteria, on the other hand, included: 1) publications not directly related to the scope of this review; 2) studies not pertaining to PCM or ncRNAs; 3) duplicates and conference materials lacking numerical data; 4) articles without available outcome data were excluded from the study. The quality of the included studies was assessed based on the size of the analyzed group, transparency of the methodology used, and completeness of the results presented. Studies with insufficient methodological descriptions or involving small sample sizes were interpreted with caution.

The methodological quality and risk of bias of the included studies were assessed using tools appropriate for the type of study. Clinical cohort studies were evaluated using the Newcastle–Ottawa Scale (NOS), which covers three domains: selection of study groups (up to four stars), comparability of groups (up to two stars), and assessment of outcomes (up to three stars). In the comparability domain, stars were awarded only if the study used multivariate survival analysis (Cox proportional hazards model) that included recognized prognostic factors; Kaplan-Meier analysis with log-rank test alone, without adjustment for confounders, was considered insufficient to award points in this domain. The total score was interpreted as high (7–9 points), moderate (4–6 points), or low (0–3 points) methodological quality (Supplementary Table S1).

Preclinical studies conducted on animal models were assessed using the SYRCLE risk of bias tool, which covers ten domains: sequence generation, baseline characteristics, allocation concealment, random housing, blinding intervention, random outcome assessment, blinding outcome, incomplete outcome data, selective outcome reporting, and other sources of bias. Each domain was classified as low risk of bias (+), high risk of bias (−), or unclear risk (?), with the “unclear risk” category used when the methodology section lacked sufficient information - for example, when randomization was mentioned but the method of sequence generation was not described. In studies combining *in vivo* and *in vitro* experiments, only the animal model part was evaluated with the SYRCLE tool (Supplementary Table S2).

Studies conducted exclusively on cell lines, as well as the *in vitro* components of combined studies, were not assessed using the SYRCLE tool, which is intended for animal research. There is no analogous scoring system for evaluating the quality of *in vitro* studies. Therefore, these data were discussed qualitatively and serve as mechanistic support with the lowest level of evidence, requiring confirmation in animal models and clinical studies.

The level of evidence was stratified by separately analyzing and discussing clinical and preclinical studies. Clinical study data were considered as higher-weight evidence regarding the value of ncRNAs as biomarkers of treatment response, while preclinical results - conducted on cell lines and animal models - were interpreted as hypothesis-generating evidence, providing mechanistic support but requiring confirmation in clinical studies. Interpretation took into account limitations arising from the methodological quality of individual studies, including small sample sizes and lack of adjustment for prognostic factors in survival analyses.

It is worth emphasizing that research on ncRNAs as biomarkers requires standardization in terms of RNA isolation methods, quantification, and data normalization. This is due to the fact that methodological variability is a significant limitation of these analyses, as it has a significant impact on the obtained expression results of molecules (42,43). This also applies to the use of ncRNAs as liquid biopsy markers in clinical practice, which requires consideration of both preanalytical and analytical factors that can significantly affect the reproducibility of results (44). Therefore, adequate quality control and standardization of assays are essential to obtain reliable analytical results.

**FIGURE 2:**
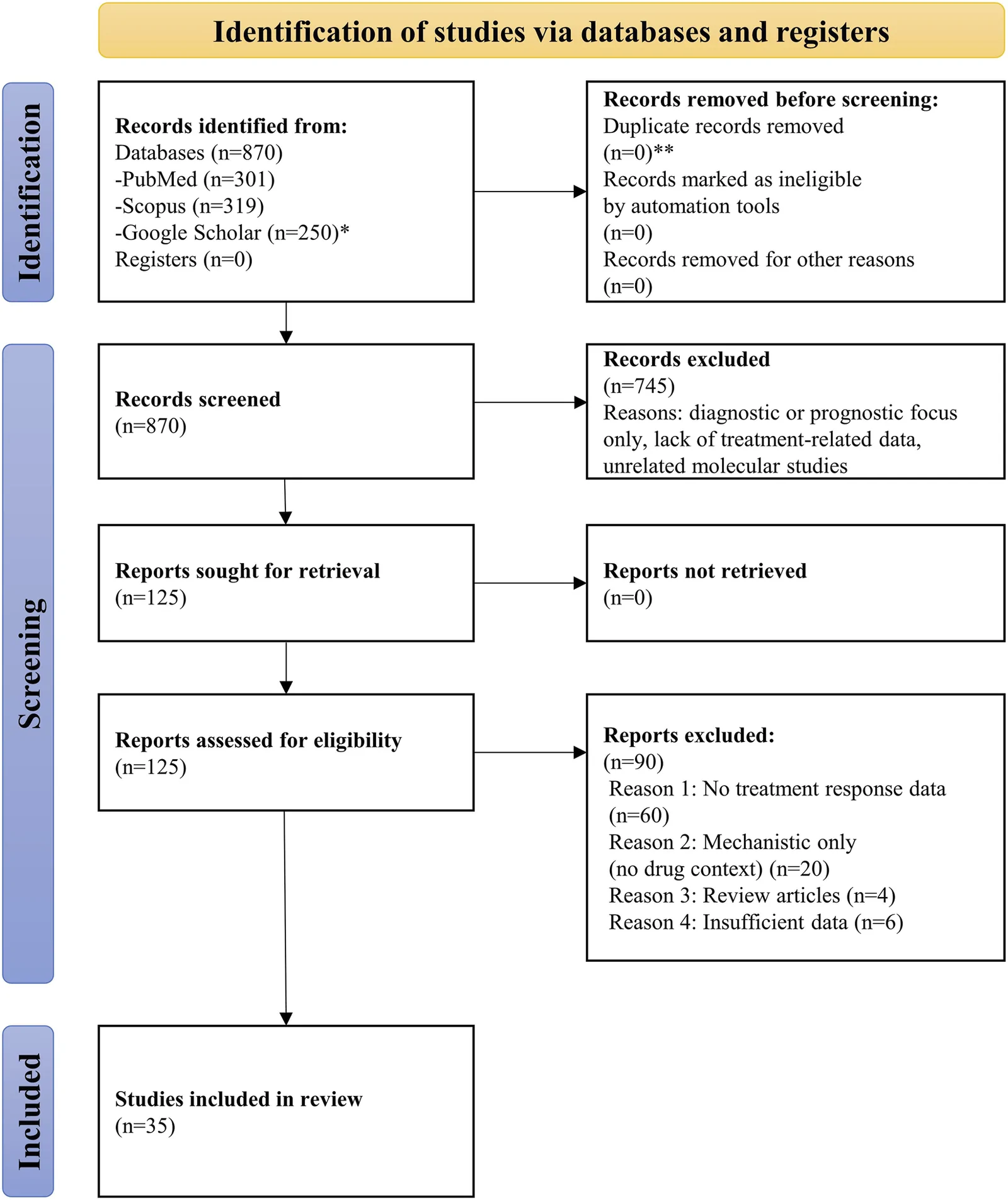
PRISMA diagram showing the strategy for selecting the included studies.

The selection process is presented in the PRISMA diagram (Figure 2): n = 870 records were identified through database searching. After removing studies that did not meet the inclusion criteria, the remaining eligible records underwent full-text assessment, and n = 35 studies were included in the analysis. Review articles were used to describe biological and clinical background, and thus were not included in the study count. Only original clinical and preclinical studies formed the evidence base for the qualitative synthesis.

The following were extracted from each cohort study: group size, study biomarker, main target(s), drug/treatment regimen, and surrogate of response (predictive value). In studies on experimental models, the following were extracted: *in vitro* and *in vivo* models, study biomarker, main target(s), drug/treatment regimen, and surrogate of response (predictive value). In this review, we describe selected ncRNAs whose expression levels are mainly associated with response to PIs and IMiDs. Parameters describing treatment response in cohort studies were defined according to the IMWG response criteria as follows: complete response (CR), very good partial response (VGPR), partial response (PR), and overall response rate (ORR). Additionally, time-to-event outcomes were assessed, including time to progression (TTP), defined as the time from treatment initiation to documented disease progression (complete data) or death unrelated to progression or last follow-up (censored data), and PFS, defined as the time from treatment initiation to disease progression or death from any cause (complete data) or last follow-up (censored data) (32). In the case of experimental models, the treatment effects were evaluated by reduced viability.

No quantitative analysis was performed owing to the diversity of the analyzed ncRNAs, therapeutic regimens, and endpoints. The results of these studies were qualitatively summarized based on the relationships between ncRNA expression and treatment response. Particular attention was paid to the consistency of the clinical and preclinical results obtained and the possible molecular mechanisms underlying the observed effects. In addition, both positive and negative results of the selected studies were included in the analysis to ensure proper interpretation of the existing evidence.

### NcRNA biomarkers for the prediction of response to PCM treatment

NcRNAs are molecules that are not translated into proteins but have important functions in cells by participating in the regulation of gene expression (from transcription, through post-transcriptional processing, to translation). Consequently, the discovery of ncRNAs has completely changed the perception of the genome, as a result of the observation that an important part of the transcribed genetic material does not encode proteins, but plays a crucial role in the regulation of biological processes and in the pathogenesis of many diseases, including cancer (45). NcRNAs are divided into two categories according to their size: short (<200 nucleotides (nt)) and long ncRNAs (≥200 nt) (46). The most important representatives of short ncRNAs are miRNAs, which bind to mRNA and induce its translation or degradation (47). Other short ncRNAs include small interfering RNA (siRNAs), piwi-interacting RNA (piRNAs) and small nucleolar RNA (snoRNAs). In contrast, lncRNAs can function as ‘sponges’ for miRNAs, are involved in the organization of chromatin structure, and also recruit protein complexes responsible for epigenetic modifications (DNA methylation, histone modifications) (48). In recent years, circRNAs, which form circular structures and can function as gene expression regulators or miRNA traps, have also been intensively investigated (49). All these types of ncRNAs contribute to the formation of an extensive regulatory network in the cell, which can be disrupted in the course of many diseases, including neurodegenerative, autoimmune, and cancerous diseases (50).

**FIGURE 3:**
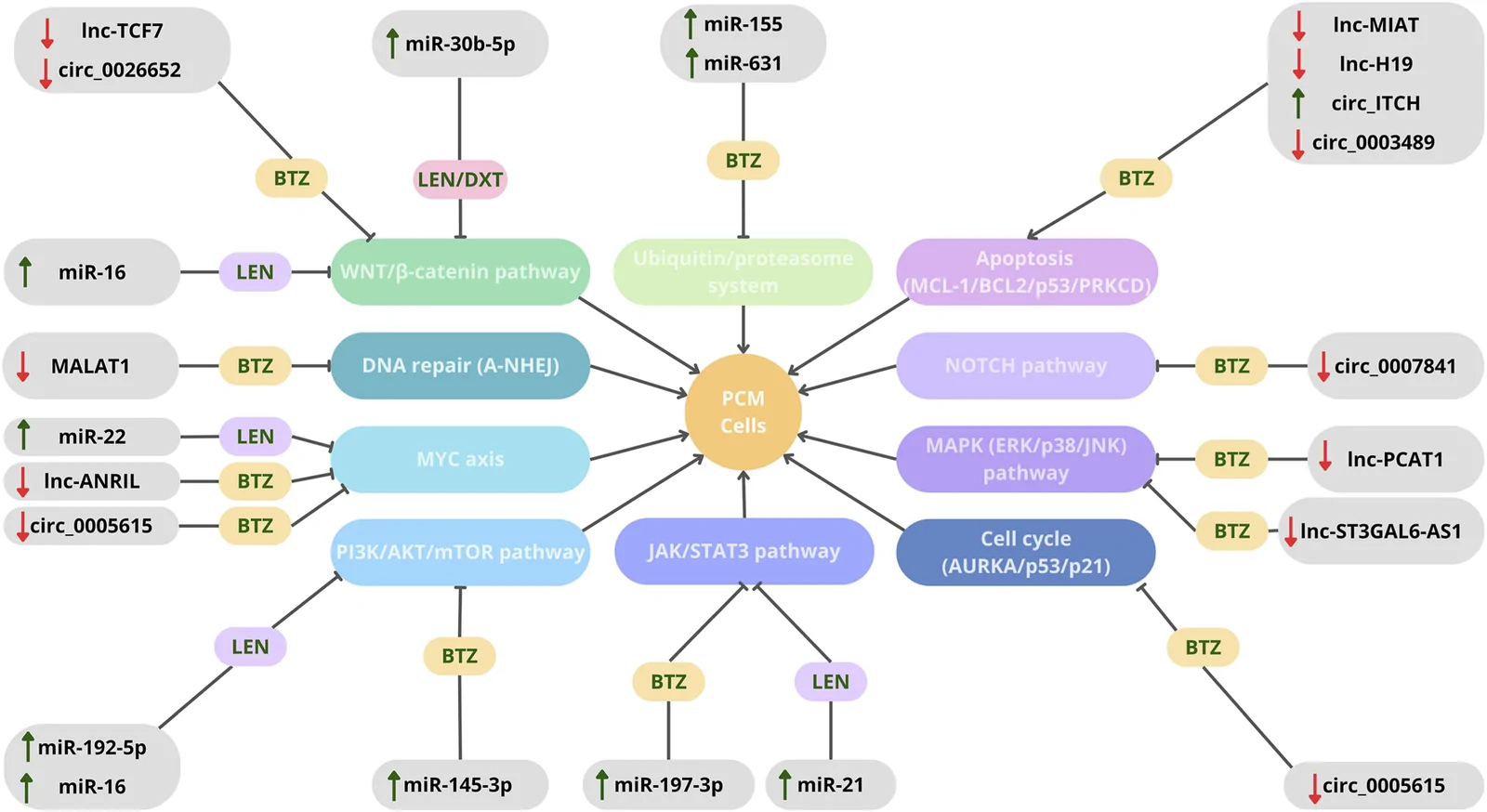
Integration of the relationship between ncRNAs, signaling pathways and drugs in the PCM. Blunt lines indicate inhibitory interactions, while arrows indicate the activating effects of a given drug within a given molecular axis. Green upward arrows and red downward arrows indicate upregulation and downregulation of individual ncRNAs, respectively, according to the information contained in the cited studies, reflecting changes in expression that lead to increased cell sensitivity to the drug. Abbreviations: AURKA, Aurora Kinase A; B, cell leukemia/lymphoma–2 – BCL2; BTZ, bortezomib; DXT, dexamethasone; JAK/STAT3, Janus kinase/signal transducer and activator of transcription 3; LEN, lenalidomide; MAPK, mitogen-activated protein kinase; MCL–1, myeloid cell leukaemia sequence 1; PCM, plasma cell myeloma; PRKCD, protein kinase C delta.

Certain ncRNAs (including lncRNAs and miRNAs) play an important role in modulating the promoter regions of genes located within areas affected by cytogenetic changes, potentially leading to their epigenetic silencing or activation. For instance, they act by recruiting chromatin-modifying complexes or influencing DNA methylation, which may enhance the activity of oncogenes or inhibit tumor suppressor genes. This regulation promotes PCM progression and is likely important for prognosis and treatment. Recently, there has been an increasing number of studies on the use of ncRNAs in the assessment of response to PCM treatment. NcRNAs influence numerous signalling pathways related to the proliferation and survival of cancer cells (Figure 3). By influencing genes involved in processes such as apoptosis or genetic repair, they can increase the effectiveness of therapy or lead to the development of resistance to a selected group of drugs (51,52). Many potential biomarkers have been identified, including miRNAs, lncRNAs, and circRNAs. However, there are still some limitations to their use in clinical practice, primarily the quality of biological material and the lack of clear correlations with therapy effectiveness. Consequently, there is a need to continuously search for new indicators that can help in the early detection of PCM recurrence, prediction of treatment effectiveness, and adaptation of therapies to the individual needs of patients.

### NcRNAs regulating apoptosis in PIs resistance

NcRNAs, including miRNAs, lncRNAs, and circRNAs, regulate core signaling pathways relevant to drug sensitivity, such as survival signaling (IL-6/Janus kinase/signal transducer and activator of transcription 3 (IL-6/JAK/STAT3), nuclear factor-κB (NF-κB)), proteostasis, DNA repair, epigenetic reprogramming, and the Wnt/β-catenin axis. Many of these pathways are central to the mechanisms of action of PIs, making ncRNAs biologically plausible as predictive biomarkers. The ncRNAs described predominantly influence epigenetic regulation and gene expression networks, including the modulation of transcription factors and signaling pathways, such as Wnt/β-catenin. However, rather than pointing to a single dominant pathway, current evidence suggests that ncRNAs act through multiple interconnected processes that collectively govern cell survival and treatment response. Synthesized findings from preclinical and clinical studies highlight both convergent mechanisms of action and important limitations, supporting the view that ncRNA-mediated regulation is complex, context-dependent, and distributed across several survival-related pathways (53). While this multifunctionality is biologically intriguing, it makes it difficult to identify a single primary mechanism, as ncRNAs interact with multiple targets simultaneously. Therefore, individual molecules have limited predictive value, and a better understanding of these relationships requires the integration of different types of data.

In the cohort studies presented (n = 60–216), conducted mainly in Asian populations (6 out of 8), bone marrow and serum material (in five and three studies, respectively) were analyzed to identify ncRNAs associated with the response to bortezomib in patients with PCM. First, changes in the expression of these promising markers under the influence of the treatment were assessed, suggesting the potential predictive value of the selected molecules. Importantly, the results indicate that better treatment outcomes correlate not so much with baseline expression levels as with a dynamic decrease in the expression of certain lncRNAs and circRNA-such as MALAT1, TCF7, ANRIL, and circ_0026652-observed after treatment with bortezomib. At the same time, numerous studies on experimental models have analyzed the relationship between ncRNAs expression and sensitivity to PIs in the context of various regulatory mechanisms. Despite the heterogeneity of the biological material and the methodologies used, these molecules exhibit a common mode of action, focusing on the regulation of cell proliferation and survival pathways. However, further studies are needed in larger groups and across different populations to determine whether the observed associations are universal or vary depending on ethnic and genetic factors. Only sufficiently large and diverse cohorts will allow for a reliable assessment of the predictive value of these molecules and reduce the risk of false-positive results.

Decreased MALAT1 expression negatively affects FOXP1, leading to inhibition of PCM cell proliferation by inducing apoptosis and arresting cancer cells in the G1/S phase. However, it stimulates SOX13, reducing tumor formation and PCM invasion (54). In addition, patients with a more significant reduction in MALAT1 expression after treatment had a longer PFS (24 months) than those with a smaller change in the expression of this molecule (11 months) (55). A similar pattern of change was observed for lnc-TCF7, which contributes to the inhibition of Wnt signaling pathways and the recruitment of the SWI/SNF complex to the *TCF7* promoter. This leads to the silencing of this gene and protection against uncontrolled cancer cell growth and disease progression, as evidenced by significantly longer PFS in patients with low expression of this lncRNA (56). ANRIL also shows reduced expression after treatment, which is associated with impaired interaction between ANRIL and EZH2, as well as the binding of H3K27me3 to the promoter region of the homologue of tenase phosphatase (PTEN) (57). This results in increased PTEN expression, which reduces the production of anti-apoptotic proteins and promotes apoptosis in PCM cells (58). In addition, it is correlated with an increased CR rate in patients (59). A similar pattern was observed for circ_0026652, whose reduced expression after treatment leads to silencing of the *WNT1* gene, which regulates the Wnt/β-catenin pathway and is responsible for the proliferation, migration, and adhesion of PCM cells to bone marrow stromal cells, as well as for the development of drug resistance. Importantly, the Wnt/β-catenin pathway is dependent on miR-608, which inhibits the expression of the frizzled four receptor, thereby inducing the oncogenic pathway. Unlike lncRNAs, circRNAs mainly act post-transcriptionally, and their effects can be integrated in the form of Wnt signaling. It is also worth mentioning that patients with reduced expression had prolonged PFS (60–62). Data from cohort studies indicate that reduced ncRNAs expression after bortezomib treatment is particularly associated with the regulation of transcriptional and epigenetic changes that enhance cell proliferation and survival. However, when interpreting the above studies, their methodological quality should also be taken into account. The highest level of evidence according to the NOS was found in the studies by Zhang, et al. (NOS 7/9) and Yin, et al. (NOS 9/9). In contrast, moderate quality was observed in the analyses by Cho, et al. and Li, et al. (NOS 5/9). It is important to emphasize that conclusions drawn from studies with lower methodological quality, therefore, require more cautious interpretation.

Despite the average cohort size, the reproducibility of individual ncRNAs in various studies remains limited. The vast majority of these markers have been identified only in single-cohort analyses, which reduces their usefulness in clinical practice.

Compared to limited cohort data, studies on cell lines and animal models consider a wider range of mechanisms through which ncRNAs may affect the sensitivity of PCM cells to bortezomib. Preclinical studies provide mechanistic justification for these clinical patterns. The strongest evidence concerns miRNAs directly modulating known resistance pathways. These include the regulation of cytokine axes, control of the cell cycle and apoptosis, and modulation of proteasome components. One of these molecules is miR-197-3p, whose overexpression causes a decrease in the expression of interleukin 6 (IL-6) and factor 6 associated with MYST/ESA1 (MEAF-6), leading to inhibition of the IL-6/JAK/STAT3 signaling pathway, which is strongly regulated in many cancer cells (53,63). In turn, miR-145-3p and miR-631 influence the response to bortezomib by modulating the key regulators of survival and apoptosis. Increased expression of miR-145-3p directly contributes to the inhibition of histone deacetylase 4 (HDAC4), contributing to increased activity of the pro-apoptotic protein B-cell leukemia/lymphoma 2-like protein 11 (BCL2L11) and inactivation of MaPCMal target of rapamycin 1 (MTORC1). This leads to the redirection of PCM cells onto the path of apoptosis and cell death caused by autophagy (64). The miR-631 molecule binds directly to the 3′UTR region of UbcH10 mRNA, inhibiting its translation and reducing the level of UbcH10 protein. In addition, a decrease in MDR1 (multiple drug-resistant protein) levels is also observed through the promotion of its ubiquitination. Thus, overexpression of miR-631 in PCM cells leads to a significant increase in cell sensitivity to bortezomib by affecting the cell cycle (65). Another molecule described is miR-155, whose mechanism is linked to the 3′UTR region of the mRNA of PSMβ5, which is the target of PIs. Increased miR-155 expression inhibited PSMβ5 expression, thereby reducing proteasome activity and increasing the sensitivity of PCM cells to bortezomib. This process indicates the possibility of direct regulation of therapeutic sensitivity, which distinguishes these ncRNAs from other miRNAs, which primarily interact through indirect signaling axes (7).

In experimental models, individual lncRNAs associated with the response to bortezomib primarily focus on the regulation of anti-apoptotic proteins and the cell cycle, as well as DNA repair mechanisms. Downregulation of MIAT significantly increased miR-29b expression, while decreasing the expression of myeloid cell leukemia sequence 1 (MCL-1), cyclin-dependent kinase 6 (CDK-6), proteasome activator subunit 4 (PSME4), and specificity protein 1 (SP-1). The results indicated that MIAT acts as a molecular sponge for miR-29b, and its downregulation increases its expression, which contributes to the inhibition of PCM development and a reduction in the expression of a well-known proliferation marker (Ki67) (66). Similarly, lncRNA H19 affects the miR-29-3p/MCL-1 axis, as well as the anti-apoptotic protein from the B-cell lymphoma 2 (BCL-2) family, which is overexpressed in many cancers (67). Reduced expression of H19 results in increased availability of miR-29-3p (a tumor suppressor), enhancing its biological activity. And miR-29-3p, responsible, among other things, for the negative regulation of MCL-1 translation, leads to increased sensitivity of PCM cells to bortezomib (68). In both cases, MCL-1 regulation is the point of convergence, indicating that lncRNAs may influence processes related to the development of PCM cell resistance by affecting survival signals. In contrast, downregulation of PCAT-1 lncRNA expression inhibits the activity of mitogen-activated protein kinase (MAPK) and JNK/p38 pathways involved in PCM cell proliferation and survival. Furthermore, reduced PCAT-1 expression leads to slowed tumor growth, decreased cell proliferation (Ki-67), and increased expression of the apoptosis marker caspase 3 (8). Of particular interest is the lncRNA metastasis associated lung adenocarcinoma transcript 1/poly-ADP-ribose polymerase-1/DNA ligase 3 (MALAT1/PARP1/LIG3) axis, in which MALAT1 participates in DNA damage repair processes by binding to the PARP1/LIG3 complex, presumably enabling the recruitment of repair proteins. Therefore, MALAT1 knockdown leads to increased apoptosis of PCM cells, while increasing the number of DNA damages and hindering their repair (defects in the nonhomologous DNA end joining (N-HEJ) repair pathway, whose main components are PARP1 in complex with LIG3) (69). Unlike the MCL-1/BCL-2 axis, this mechanism focuses on genomic stabilization and the response to DNA damage.

Unlike lncRNAs, which mainly interact at the chromatin level, circRNAs primarily exert post-transcriptional effects, although their functional impact may converge at similar regulatory nodes. The circRNAs, due to their circular structure and the presence of multiple binding sites, are commonly responsible for absorbing miRNA, functioning as a ‘molecular sponge’ to regulate the expression of various molecular targets for specific miRNAs. In addition, they can directly interact with proteins, leading to the control of their transcription and translation (70–72). Circ_0005615 is associated with the regulation of the miR-185-5p/interferon regulatory factor 4 (IRF4) axis. Increased expression of circ_0005615 likely leads to the blocking of miR-185-5p activity by acting as a “molecular sponge,” thereby inhibiting the negative regulation of IRF4, whose elevated levels are associated with bortezomib resistance. Silencing circ_0005615 increases the sensitivity of PCM cells to therapy and contributes to tumor growth inhibition (73). Similarly, circ_0007841 acts *via* miR-129-5p on the expression of the surface ligand Jagged 1 (JAG1), which is a part of the Notch signaling pathway. Silencing circ_0007841 leads to a decrease in JAG1 expression *via* an increase in miR-129-5p levels, which inhibits proliferation and induces PCM cell sensitivity to bortezomib therapy (9). Hsa_circ_0003489 has been designated as a regulator of the miR-874-3p/histone deacetylase 1 (HDAC1) axis. Silencing hsa_circ_0003489 caused it to cease functioning as a molecular sponge for miR-874-3p. In turn, overexpression of this molecule inhibits HDAC1 expression, leading to PCM cell apoptosis (74,75). Another circRNA molecule that may be relevant to the efficacy of bortezomib in PCM is circITCH. It acts *via* miR-615-3p on its target, protein kinase C delta (PRKCD), which is involved in stress response and apoptosis control. Increased expression of circITCH may act as a molecular sponge for miR-615-3p, which is usually overexpressed in PCM cells, and subsequently results in a decrease in PRKCD levels (76). Despite the impact of the described circRNAs on various molecular targets, their common feature is the regulation of important control points of the plasma cell phenotype and the mechanisms of adaptation to therapy. However, despite the strong mechanistic rationale, most circRNA data are limited to single-cell models, underscoring the need for clinical validation.

Studies conducted on cell lines only partially reflect complex tissue interactions, the influence of the microenvironment, and the systemic response. In contrast, animal models capture these relationships more fully. However, interspecies differences in ncRNA expression make it difficult to directly translate results to humans. An additional limitation is the variation in ncRNAs stability between experimental models and patient biological samples.

### NcRNAs modulating immune pathways in IMiDs response

Cohort studies (n = 48) of moderate methodological quality (NOS 5/9) indicated that lenalidomide therapy effectiveness is associated with changes in miR-16 and miR-21 expression. Higher levels of miR-16 expression lead to the inhibition of PCM cell proliferation by modulating the activity of AKT3, MAP kinases, and the NF-κB/MAP3KIP3 activator. This process is associated with the reduced expression of genes promoting cell proliferation, such as *VEGF*, cyclin D1 (*CCND1*), *WNT3A*, and *MCL-1*, indicating the involvement of miR-16 in enhancing the response to treatment. In turn, lower miR-21 expression affects the JAK/STAT3 and NF-κB axes and may lead to the reduction of uncontrolled growth, proliferation, and migration of PCM cells through decreased expression of anti-apoptotic genes such as *MCL-1*, *BCL-XL*, and *BCL-2*. Notably, higher miR-16 expression may promote a better response to treatment, exceeding the PR level. In contrast, lower miR-21 expression is associated with a deeper response to treatment (≥VGPR) (53,77,78). Despite differences in their direct molecular targets, both miR-16 and miR-21 influence the regulation of the NF-κB axis and MCL-1.

In experimental models, the response to lenalidomide has been linked to miRNAs responsible for regulating key proliferation and survival points, including transcription factors and DNA repair mechanisms. MiR-22 acts as a regulator of the MYC-dependent axis by directly binding to the mRNA of this transcription factor (in the 3′UTR and 5′UTR regions). Overexpression of miR-22 leads to the inhibition of translation and reduction of MYC activity, which promotes apoptosis of PCM cells and sensitizes them to lenalidomide (79). In turn, overexpression of miR-192-5p causes a decrease in both PARP1 expression and AKT signaling pathway activity. This indicates dual regulation by miR-192-5p in processes such as the response to genetic damage and control of cell survival (80). These data indicate that the effectiveness of therapy may depend on the balance between proliferative pathways and DNA repair mechanisms. These pathways align with the core mechanisms of IMiDs (immunomodulation, oxidative stress, and interference with transcription factors).

### NcRNAs in combination regimens

In cohort analyses of patients (n = 62–83) who underwent combination therapy (lenalidomide/dexamethasone and lenalidomide/bortezomib/dexamethasone), changes in ncRNAs expression associated with the regulation of WNT signaling, angiogenesis and stress-dependent protein kinases were observed. In the lenalidomide/dexamethasone treatment regimen, increased expression of miR-30b -5p contributes to a reduction in vascular endothelial growth factor (VEGF) and B-cell CLL/lymphoma 9 (BCL9) protein levels, and consequently to the inhibition of angiogenesis and PCM cell proliferation and migration by limiting the activation of the Wnt signaling pathway, which mediates tumor development. It is worth noting that miR-30b-5p overexpression is strongly associated with a good response and is linked to prolonged TTP in PCM patients (81,82). However, when lenalidomide/bortezomib/dexamethasone is used, reduced expression of lncRNA PCAT-1 is observed, which acts on the *NIMA* gene, associated with cell cycle regulation, and affects PCM cell apoptosis. In addition, reduced expression of this lncRNA affects the MAP3K7/NF-κB signaling pathways associated with stress response and inflammation. The expression of PCAT-1 prior to treatment is significantly lower in patients who subsequently achieved CR compared to those who did not. Furthermore, high PCAT-1 expression was significantly correlated with poorer PFS (HR = 2.38) (83,84). Importantly, both analyses have a high level of methodological quality (respectively; NOS 7/9 and NOS 9/9).

In a study on experimental models treated with bortezomib/dexamethasone/doxorubicin, miR-21 expression is reduced by inhibiting the NF-κB pathway, which lead to the induction of suppressor genes such as *PTEN* and *PDCD4* and promoted PCM cell apoptosis. Furthermore, inhibition of miR-21 expression contributes to increased sensitivity to dexamethasone and doxorubicin (85). In turn, in experimental models treated with carfilzomib/bortezomib/melphalan, lncRNA ST3GAL6-AS1 is associated with the regulation of the MAPK pathway, which plays an important role in integrating both proliferative and stress signals. It was also associated with the expression of Ras-related protein 1 (RAP1), which is responsible for controlling adhesion and responding to external stimuli. Silencing lncRNA ST3GAL6-AS1 leads to a reduction in the number of polyubiquitinated proteins, which can weaken the action of proteasome inhibitors. Consequently, reducing its expression does not increase the sensitivity of cells to treatment, but rather acts antagonistically to bortezomib and carfilzomib. Therefore, combining these drugs to target the lncRNAs may be disadvantageous, and cells may activate alternative signaling pathways for survival (86).

Despite the growing number of reports, evidence supporting the role of ncRNAs in the therapeutic response of PCM remains limited. Cohort studies usually involve small groups of patients, while most data come from preclinical models based on cell lines and animal models. In addition, molecular heterogeneity and diverse therapeutic regimens make it difficult to interpret the results. In combination therapy, ncRNAs influence both survival pathways and the mechanisms regulating mitosis and cellular stress responses, demonstrating the complexity of cellular adaptation to the action of drugs. Therefore, further studies in large populations are needed to reliably assess the clinical potential of these molecules (87).

## Discussion

The data collected in this review indicate the predictive potential of selected miRNAs (miR-30b-5p, miR-16, miR-21), lncRNAs (MALAT1, TCF7, ANRIL, PCAT-1) and circRNAs (circ_0026652) in cohort studies of PCM patients, as well as ncRNAs analyzed in experimental models (miRNAs: miR-197-3p, miR-145-3p, miR-155, miR-631, miR-22, miR-192-5p; lncRNAs: MIAT, H19, PCAT-1, MALAT1, ST3GAL6-AS1; circRNAs: circ_0005615, circ_0003489, circ_0007841, circITCH). These molecules are significantly associated with the regulation of plasma cell proliferation and survival, and their predictive value stems from their influence on common signaling axes, i.e., NF-κB, IL-6/JAK/STAT3, Wnt/β-catenin, and MAPK.

A key insight from clinical cohorts is that differences in ncRNAs expression between pre- and post-treatment during therapy are more informative than baseline levels. The consistent observation that decreases in MALAT1, ANRIL, TCF7, and circ_0026652 after bortezomib treatment correlated with deeper responses supports their potential role as early treatment response indicators rather than static markers of risk. An exception is the lenalidomide/dexamethasone therapy study. In this case, the predictive value was based on the baseline level rather than the change over time (8,55,56,59,60,77,81).

Based on this review, attention should be paid to miR-21, which is considered one of the best-known oncomirs (88). Wang et al. also showed that miR-21 contributes to the increased resistance of PCM cells to treatment with bortezomib, dexamethasone, and doxorubicin (85). Among the lncRNAs studied in PCM patient cohorts and experimental models, MALAT1 has been the best characterized. Its overexpression has been associated with increased proliferation and poor prognosis (89). Therefore, MALAT1 inhibition increases the sensitivity of PCM cells to bortezomib by modulating the expression of survival genes, as demonstrated by Cho et al. (55). However, elevated MALAT1 levels have also been observed during treatment with bortezomib and doxorubicin (90). These results indicate that MALAT1 might serve as a predictive marker, however this requires further research. Similar to MALAT1, PCAT-1 is also one of the well-described lncRNA in PCM. The convergence of data on survival and treatment response indicates significant predictive potential, but broad validation in independent populations is needed to apply PCAT-1 in clinical practice (8,83). In the context of response to therapy, Zhao et al. observed reduced expression after treatment with bortezomib/cyclophosphamide/dexamethasone and bortezomib/lenalidomide/dexamethasone (83).

Other molecules described in cohort studies include miR-16, which is a cell cycle regulator and may act as a suppressor of the tumorigenic process by influencing BCL2 (91). Hao et al. showed that increased miR-16 expression occurs after treatment with bortezomib and melphalan, whereas it decreases as a result of fluctuations in IL-6 levels (92). Another ncRNAs analyzed in patient cohorts was miR-30b-5p. It belongs to the miR-30 family, which participates in the regulation of differentiation and response to cellular stress. Jung et al. observed that abnormalities in the expression of this miRNA family affect the therapeutic response in patients with PCM. Elevated expression of both miR-30b-5p and miR-30c-5p is associated with a good response to lenalidomide/dexamethasone (81). In turn, overexpression of miR-30e-5p has also been demonstrated in PCM patients with gain of 1q21 (93). Therefore, to correctly interpret the level of ncRNAs expression, the clinical context must be considered (94). In contrast, overexpression of ANRIL in PCM cells results in *PTEN* gene repression, which causes the development of bortezomib resistance and poor prognosis. Furthermore, polymorphisms in the *ANRIL* gene contribute to cancer recurrence after ASCT. However, changes in its expression occur not only in PCM but also in other cancers, such as colorectal and prostate cancer (95,96). Studies by Ding et al. and Liu et al. have shown that reduced TCF7 expression occurs in patients with CR, and overexpression predicts poorer OS (97,98). Circ_0026652 is also a potential predictive marker. Its reduced expression increases the sensitivity of cells to bortezomib, whereas overexpression contributes to poor response to therapy and worse PFS and OS in patients with PCM (60).

Overall, the most reliable evidence originates from higher-quality cohort studies with higher NOS scores and is further strengthened by concordant findings from preclinical models. In contrast, results from single-cohort studies or analyses without multivariable adjustment should be considered as exploratory.

Analyses of cell lines and animal models provide justification for the mechanisms underlying the observed clinical correlations and allow for the modification of the expression of the studied ncRNAs and assessment of their changes. However, their use in clinical practice requires further validation. All miRNAs, including miR-197-3p, miR-145-3p, miR-155, miR-631, miR-22, and miR-192-5p, showed increased expression after treatment. The increase in the expression of these ncRNAs after therapy may reflect the activation of pro-apoptotic processes, which is consistent with the mechanism of effective drug action in the course of PCM (98,99). Conversely, miR-155, which is classified as an oncomir that promotes cell survival, may also be upregulated after therapy. This phenomenon is likely to be an adaptation of cancer cells to treatment-related stress (100). In turn, miR-631, miR-22, and miR-192-5p are associated with the regulation of the cell cycle and response to DNA damage by modulating the function of repair proteins and cycle checkpoints, which may affect the sensitivity of cells to therapies (65,79,101). Studies on H19 and MIAT emphasize their role in regulating PCM cell proliferation and growth, but at the same time point to the small number of studies linking the expression of these ncRNAs to therapeutic response in patient cohorts (66,67). ST3GAL6-AS1 is an lncRNA overexpressed in PCM cells; therefore, silencing ST3GAL6-AS1 leads to the inhibition of adhesion, migration, and invasion of cancer cells. This is related to the effect of ST3GAL6-AS1 on the bone marrow microenvironment, but there are no analyses in large patient cohorts in the literature (101). The same applies to circRNAs. Circ_0005615, circ_0003489, circ_0007841, and circITCH are described as “miRNA sponge” regulators that affect the proliferation and survival axes by sequestering specific miRNAs. Reviews on circRNAs in PCM indicate their potential predictive value, while noting the lack of specific clinical data (102).

The data presented indicate that ncRNAs in PCM do not act as single regulatory molecules but as elements of an integrated network controlling the function of plasma cells. This may suggest that their predictive value results from the location of individual ncRNAs in a larger signaling network that determines the response to the therapy. The most promising ncRNAs include miR-21, lncRNA MALAT1, and PCAT-1, which have been widely described in the context of the molecular mechanisms and clinical observations in patients with PCM. The results of these analyses showed consistency between the cohort observations and *in vitro* functional data. This consistency supports prioritizing these ncRNAs for prospective translational validation, rather than immediate clinical implementation. Unlike the ncRNAs mentioned above, the remaining ncRNAs are mainly based on experimental models. Data from such studies focus on the mechanisms of action of ncRNAs in drugs used in PCM therapy. However, they do not reflect, among other things, the heterogeneity of PCM, which is crucial in the development of resistance to treatment. Therefore, cellular data are insufficient to qualify a given molecule as a reliable predictive indicator.

The main limitations associated with this study should also be noted. First, methodological heterogeneity results from differences in biological materials (bone marrow, plasma, cell lines, and animal models), measurement techniques, and endpoints, making it difficult to compare results (103). Moreover, several ncRNAs have demonstrated conflicting patterns (e.g., MALAT1 increases during some treatments, decreases in others). Such discrepancies likely reflect the timing of sampling, treatment context, and biological heterogeneity, emphasizing that predictive biomarkers require context-specific interpretation. In addition, most cohort studies involved a small number of patients and were retrospective in nature. Furthermore, there is a lack of independent validation in large, prospective, multicenter studies, and very few studies have integrated ncRNA markers with established clinical tools (R2-ISS, cytogenetics, and MRD status). Finally, although numerous preclinical models have identified mechanistically compelling miRNAs and lncRNAs, these findings often rely on a narrow set of cell lines. It should also be noted that the publications included in this review demonstrated considerable variation in methodological quality. For instance in some of the clinical studies, control of important and additional factors were not applied, which weakens the conclusions regarding independent prognostic value. In contrast, in most of the preclinical studies, the risk of bias assessment revealed an unclear risk in the areas of randomization and blinding. The complexity of patient tumors, which are genomically and microenvironmentally heterogeneous, remains a major challenge. Therefore, even the best-characterized molecules cannot be used to predict the response to PCM treatment.

In summary, the results presented here indicate the potential of ncRNAs as predictive biomarkers of PCM. However, only selected molecules demonstrating robust biological plausibility supported by multidimensional evidence, i.e., miR-21 (one of the most extensively validated oncomirs in PCM; linked to both IL-6/JAK/STAT3 and NF-κB signaling), MALAT1 (supported by *in vitro*, *in vivo* and cohort data; functionally connected to DNA repair *via* PARP1/LIG3), and PCAT-1 (consistently downregulated in responders to VRd and mechanistically linked to MAPK/NF-κB and cell cycle regulation), may be considered as potential candidates for future clinical development. Future studies should focus on combining ncRNAs profiles with clinical data and prospective validation. Only such an approach will enable the transition from molecularly justified candidates to real predictive tools that are helpful in the treatment of PCM.

A summary of the biomarkers discussed above is presented in Table 1 and 2.

**TABLE 1:** NcRNAs potentially relevant for assessing response to chemotherapy in PCM (studies on experimental models).

Authors (year)	*In vitro* model	*In vivo* model	Study biomarker	Main target(s)	Drug/treatment regimen	Surrogate of response (predictive value)
Liu, et al. (2022)	RPMI8226, H929, U266, KMS11, KMS26	Xenograft tumor model in NOD/SCID female mice	miR-197-3p	MEAF-6, IL-6/JAK/STAT3 signaling pathway	BTZ	Upregulation^a^ related to inhibition of IL-6/JAK/STAT3, reduced viability
Wu, et al. (2020)	U266, NCI-H929, RPMI8226, LP-1, KM3	Xenograft tumor model in BALB/C nude mice	miR-145-3p	HDAC4	BTZ	Upregulation^a^ related to decreased histone deacetylase 4, reduced viability
Xi, et al. (2017)	U266, NCI-H929, RPMI8226	N/a	miR-631	UbcH10	BTZ	Upregulation^a^ related to decreased level of UbcH10 protein, reduced viability
Amodio, et al. (2019)	AMO-bortezomib	Xenograft tumor model in CB-17 SCID mice	miR-155	PSMβ5	BTZ	Upregulation^a^ related to decreased proteasome activity, reduced viability
Caracciolo, et al. (2021)	AMO1, R8226, U266, U266 MYC+, ABZB, OPM2LenRes	N/a	miR-22	*MYC*	LEN	Upregulation^a^ related to decreased MYC activity, reduced viability
Luo, et al. (2024)	NCI-H929	N/a	miR-192-5p	PARP1	LEN	Upregulation^a^ related to decreased PARP1 expression, reduced viability
Wang, et al. (2011)	RPMI8226, U266, KM3	N/a	miR-21	NF-κB signaling pathway	BTZ/DXT/DOX	Downregulation^a^ related to inhibition of NF-κB, reduced viability
Fu, et al. (2019)	U266, KMS12, KM3, U266/BTZ	Xenograft tumor model in mice	lncRNA MIAT	miR-29b, MCL-1	BTZ	Downregulation^a^ related to increased miR-29b expression and decreased MCL-1 expression, reduced viability
Pan, et al. (2019)	H929, U266, 8226	Xenograft tumor model in BALB/C male athymic mice	lncRNA H19	miR-29-3p, MCL-1	BTZ	Downregulation^a^ related to increased miR-29-3p expression and decreased MCL-1 expression, reduced viability
Shen, et al. (2019)	NCI-H929, RPMI8226, U266	Xenograft tumor model in BALB/C nude female mice	lncRNA PCAT-1	MAPK, JNK/p38 signaling pathway	BTZ	Downregulation^a^ related to decreased MAPK and JNK/p38 activity, reduced viability
Hu, et al. (2018)	H929, MM.1S, RPMI8226	N/a	lncRNA MALAT1	MALAT1/PARP1/LIG3	BTZ	Downregulation^a^ related to decreased PARP1 and LIG3 expression, reduced viability
Ronchetti, et al. (2020)	NCI-H929, LP-1, U266, AMO1	N/a	lncRNA ST3GAL6-AS1	RAP1, MAPK signaling pathway	BTZ/CFZ/Melph	Downregulation^a^ related to decreased MAPK activity and RAP1 expression, reduced viability
Fu, et al. (2022)	NCI-H929, RPMI8226	Xenograft tumor model in NOD/SCID mice	circRNA_0005615	miR-185-5p, IRF4	BTZ	Downregulation^a^ related to increased miR-185-5p and decreased IRF4 expression, reduced viability
Wang, et al. (2020)	OPM2, NCI-H929 (CRL-9068)	Xenograft tumor model in BALB/C nude mice	circRNA_0007841	miR-129-5p, JAG1	BTZ	Downregulation^a^ related to increased miR-129-5p expression and decreased JAG1 expression, reduced viability
Tian, et al. (2021)	MM1.R	Xenograft tumor model in nude mice	circRNA_0003489	miR-874-3p, HDAC1	BTZ	Downregulation^a^ related to increased miR-874-3p expression and decreased HDAC1 expression, reduced viability
Liu, et al. (2020)	U266, NCI-H929, RPMI8226	Xenograft tumor model in BALB/C nude mice	circRNA ITCH	miR-615-3p, PRKCD	BTZ	Upregulation^a^ related to decreased miR-615-3p and increased PRKCD expression, reduced viability

Abbreviations: ABZB, AMO1 bortezomib–resistant; CFZ, carfilzomib; DOX, doxorubicin; DXT, dexamethasone; HDAC14, histone deacetylase 1,4; IL-6/JAK/STAT3, interleukin 6/Janus kinase/signal transducer and activator of transcription 3; IRF4, interferon regulatory factor 4; JAG1, Jagged 1; LEN, lenalidomide; LIG3, DNA ligase 3; MALAT1, metastasis associated lung adenocarcinoma transcript 1; MAPK, mitogen-activated protein kinase; MEAF-6, MYST/ESA1-associated factor 6; Melph–melphalan; MCL-1, myeloid cell leukaemia sequence 1; NF-κB, nuclear factor-κB; PARP1, poly–ADP–ribose polymerase–1; PCM, plasma cell myeloma; PRKCD, protein kinase C delta; PSMβ5, proteasome subunit β5; RAP1, Ras-related protein one.

^a^ Change in expression between pre-treatment and post-treatment time points.

**TABLE 2:** NcRNAs potentially relevant for assessing response to chemotherapy in PCM (cohort studies).

Authors (year)	Study group (n)	Study biomarker	Main target(s)	Drug/treatment regimen	Surrogate of response (predictive value)
Jung, et al. (2017)	62 RRPCM	miR-30b-5p	*BCL9*	LEN/DXT	Upregulation^a^ associated with better response and longer TTP
Gkioka, et al. (2024)	63 (48 PCM, 15 healthy)	miR-16	*VEGF, CCND1, WNT3A, MCL-1*	LEN	Upregulation^b^ correlated with higher PR rate
Gkioka, et al. (2024)	63 (48 PCM, 15 healthy)	miR-21	JAK/STAT3 signaling pathway	LEN	Downregulation^b^ correlated with higher VGPR rate
Cho, et al. (2014)	124 (104 PCM, 20 healthy)	lncRNA MALAT1	*FOXP1* (Leng, et al. (2022))	BTZ	Downregulation^b^ associated with better response (CR/VGPR/PR) and longer PFS
Zhang, et al. (2020)	276 (216 PCM, 60 healthy)	lncRNA TCF7	*TCF7*	BTZ	Downregulation^b^ associated with better response (CR) and longer PFS
Yin, et al. (2021)	107 (87 PCM, 30 healthy)	lncRNA ANRIL	*PTEN* (Yang, et al. (2021))	BTZ	Downregulation^b^ associated with better response (CR) and longer PFS
Zhao, et al. (2021)	83 PCM	lncRNA PCAT-1	*NIMA*	LEN/BTZ/DXT	Downregulation^b^ associated with a higher CR/ORR rate and longer PFS
Li, et al. (2022)	60 PCM	circRNA_0026652	*WNT1* (Spaan, et al. (2018))	BTZ	Downregulation^b^ associated with a higher CR/ORR rate and longer PFS

Abbreviations: BTZ, bortezomib; CR, complete response; DXT, dexamethasone; JAK/STAT3, Janus kinase/signal transducer and activator of transcription 3; LEN, lenalidomide; ORR, overall response rate; PCM, plasma cell myeloma; PFS, progression-free survival; PR, partial response; RRPCM, relapsed/refractory multiple myeloma; TTP, time to progression; VGPR, very good partial response.

^a^ Expression level analyzed exclusively at baseline.

^b^ Change in expression between pre-treatment and post-treatment time points.

## Conclusion

The evidence synthesized in this review demonstrates that dysregulation of selected ncRNAs, particularly miR-21, MALAT1, and PCAT-1 is repeatedly associated with responses to core PCM therapies. Notably, differences in ncRNAs expression between pre- and post-treatment appear to be more reliable than baseline expression, highlighting their potential role in the real-time monitoring of therapeutic efficacy.

Despite promising biological and clinical signals, the translational use of ncRNAs is limited by methodological heterogeneity, small cohort sizes, lack of standardization, and insufficient external validation. Only a few molecules show convergence of evidence from cohorts, mechanistic studies, and treatment-specific associations.

Based on current knowledge, the most promising predictive biomarker candidates are:

– miR-21: Mechanistically and clinically linked to resistance to lenalidomide and PIs; integrates IL-6/JAK/STAT3 and NF-κB pathways.
– MALAT1: Supported by consistent functional data connecting it to DNA repair, proliferation, and PIs response.
– PCAT-1: Reproducibly associated with CR, PFS and key survival pathways in patients treated with VRd.

To move these ncRNAs toward clinical utility, several steps are required:

1. Prospective validation in large, uniformly treated cohorts with standardized response criteria.
2. Standardization of pre-analytical (sample type, timing, storage) and analytical protocols (RNA isolation, normalization strategies).
3. Integration with existing prognostic tools, such as R2-ISS, cytogenetics, and MRD assessment.
4. Analysis of longitudinal expression patterns to distinguish dynamic predictive markers from static prognostic markers.
5. Multi-omic models combining ncRNAs with transcriptomic and proteomic data can be used to refine therapeutic stratification.

Such a structured approach offers a realistic path from experimental findings to clinically actionable predictive biomarkers capable of guiding personalized therapy in PCM.
