# Supplementary material for: Non-coding RNAs as predictive factors of oncological outcomes in plasma cell myeloma
Source: Oncol Rev. 2026 Aug 14;20:1868835. doi: 10.3389/or.2026.1868835 (PMC13522884; doi:10.3389/or.2026.1868835)
Supplement: Supplementary file 1 [file Table1.DOCX]

Supplementary Table 1. Methodological quality of studies included in the final analysis based on the Newcastle–Ottawa Scale* to assess the quality of cohort studies (n=7).

| References | Selection | | | | Comparability | Outcome | | | Total |
| --- | --- | --- | --- | --- | --- | --- | --- | --- | --- |
|  | Representativeness of the exposed cohort | Selection of the nonexposed cohort | Ascertainment of exposure | Outcome of interest was not present at the start of the study | Control of important and additional factors | Assessment of outcome | Follow-up long enough for outcomes to occur | Adequacy of follow-up of cohorts |  |
| Jung, et al. (2017) | ★ | ★ | ★ | ★ | - | ★ | ★ | ★ | 7 |
| Gkioka, et al. (2024) | ★ | ★ | ★ | - | - | ★ | ★ | - | 5 |
| Cho, et al. (2014) | - | - | ★ | - | ★★ | ★ | ★ | - | 5 |
| Zhang, et al. (2020) | ★ | ★ | ★ | ★ | - | ★ | ★ | ★ | 7 |
| Yin, et al. (2021) | ★ | ★ | ★ | ★ | ★★ | ★ | ★ | ★ | 9 |
| Zhao, et al. (2021) | ★ | ★ | ★ | ★ | ★★ | ★ | ★ | ★ | 9 |
| Li, et al. (2022) | ★ | - | ★ | ★ | - | ★ | - | ★ | 5 |

^*^Each study can be awarded a maximum of 1 star for each numbered item within the selection and exposure categories, and a maximum of 2 stars for the comparability category.
