# Supplementary material for: Non-coding RNAs as predictive factors of oncological outcomes in plasma cell myeloma
Source: Oncol Rev. 2026 Aug 14;20:1868835. doi: 10.3389/or.2026.1868835 (PMC13522884; doi:10.3389/or.2026.1868835)
Supplement: Supplementary file 2 [file Table2.DOCX]

Supplementary Table 2. Methodological quality of studies included in the final analysis based on the SYRCLE’s risk of bias tool* to assess the quality of animal studies (n=11).

| References | Sequence generation | Baseline Characteristics | Allocation Concealment | Random Housing | Blinding Intervention | Random Outcome Assessment | Blinding Outcome | Incomplete Outcome Data | Selective Outcome Reporting | Other Sources of Bias |
| --- | --- | --- | --- | --- | --- | --- | --- | --- | --- | --- |
| Liu, et al. (2022) | ? | + | ? | ? | ? | ? | ? | + | + | + |
| Wu, et al. (2020) | ? | + | ? | ? | ? | ? | ? | + | + | + |
| Amodio, et al. (2019) | ? | + | ? | ? | ? | ? | ? | + | + | + |
| Fu, et al. (2019) | ? | + | ? | ? | + | ? | ? | + | + | + |
| Pan, et al. (2019) | ? | + | ? | ? | ? | ? | ? | + | + | - |
| Shen, et al. (2019) | ? | + | ? | ? | ? | ? | ? | ? | ? | ? |
| Fu, et al. (2022) | ? | + | ? | ? | ? | ? | ? | + | + | + |
| Wang, et al. (2020) | ? | + | ? | ? | ? | ? | ? | + | + | + |
| Tian, et al. (2021) | ? | + | ? | ? | ? | ? | ? | + | + | + |
| Liu, et al. (2020) | ? | + | ? | ? | ? | ? | ? | + | + | + |

*(+) indicates low risk of bias; (-) indicates high risk of bias; (?) indicates unclear risk of bias.
